# Rapid evolution of microbe-mediated protection against pathogens in a worm host

**DOI:** 10.1038/ismej.2015.259

**Published:** 2016-03-15

**Authors:** Kayla C King, Michael A Brockhurst, Olga Vasieva, Steve Paterson, Alex Betts, Suzanne A Ford, Crystal L Frost, Malcolm J Horsburgh, Sam Haldenby, Gregory DD Hurst

**Affiliations:** 1 Institute of Integrative Biology, University of Liverpool, Liverpool, UK; 2 Department of Zoology, University of Oxford, Oxford, UK; 3 Department of Biology, University of York, York, UK

## Abstract

Microbes can defend their host against virulent infections, but direct evidence for the adaptive origin of microbe-mediated protection is lacking. Using experimental evolution of a novel, tripartite interaction, we demonstrate that mildly pathogenic bacteria (*Enterococcus faecalis*) living in worms (*Caenorhabditis elegans*) rapidly evolved to defend their animal hosts against infection by a more virulent pathogen (*Staphylococcus aureus*), crossing the parasitism–mutualism continuum. Host protection evolved in all six, independently selected populations in response to within-host bacterial interactions and without direct selection for host health. Microbe-mediated protection was also effective against a broad spectrum of pathogenic *S. aureus* isolates. Genomic analysis implied that the mechanistic basis for *E. faecalis*-mediated protection was through increased production of antimicrobial superoxide, which was confirmed by biochemical assays. Our results indicate that microbes living within a host may make the evolutionary transition to mutualism in response to pathogen attack, and that microbiome evolution warrants consideration as a driver of infection outcome.

## Introduction

Microbes can have effects on host biology far beyond their core impacts on digestion (Dillon *et al.*, 2000; Cerf-Bensussan and Gaboriau-Routhiau, 2010; Brucker and Bordenstein, 2013; Lize *et al.*, 2014). Microbes can cause infectious disease, but they can also act to protect hosts from pathogens, a phenomenon observed in a range of animals (Dillon *et al.*, 2005; Dong *et al.*, 2009; Jaenike *et al.*, 2010; Koch and Schmid-Hempel, 2011), including humans (Kamada *et al.*, 2013), and in plants at the root–soil interface (Mendes *et al.*, 2011; May and Nelson, 2014). These protective microbes provide an important complement to the host's defence systems (Abt and Artis, 2013; Hooper *et al.*, 2012; McFall-Ngai *et al.*, 2013). As pathogens invade the host, they can not only be targeted by the host immune system, but also interact with pathogenic and commensal microbial species already present within the host (McFall-Ngai *et al.*, 2013). Resident microbes can therefore provide strong protection against virulent pathogens, and corresponding microbial traits might be evolutionarily advantageous. Evolution of this nature would represent microbes evolving along the parasitism–mutualism continuum (Chamberlain *et al.*, 2014).

The large population sizes and short generation times of microbes also create the potential for the rapid evolution of such defences. Can microbes evolve to protect their host in response to virulent pathogen challenge, and, in doing so make an evolutionary transition to mutualism? It is well established that infecting pathogens can undergo rapid adaptation (Brockhurst and Koskella, 2013) in response to transmission opportunity and mode (Messenger *et al.*, 1999), prior immune exposure (Mackinnon and Read, 2004) and multi-strain coinfection (Garbutt *et al.*, 2011) with host defences known to reciprocally evolve to pathogen adaptation (Schulte *et al.*, 2010; Morran *et al.*, 2011). Despite this, evolutionary responses by resident microbes against pathogen infection have not before been considered.

**Figure 1:**
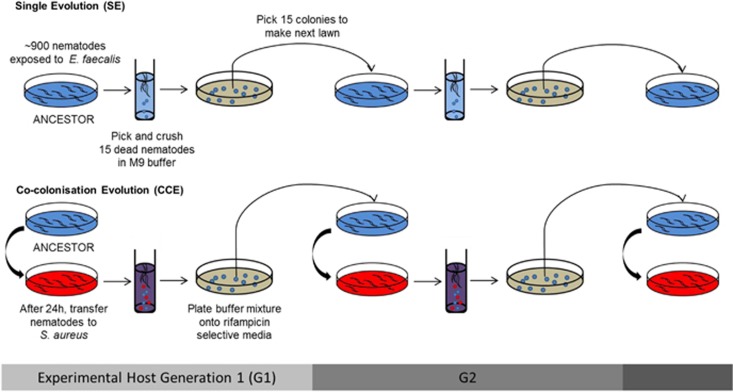
Experimental procedure for experimental evolution of *E. faecalis* within *C. elegans* populations. Treatments are shown for a single replicate population. Six populations of *E. faecalis* were independently passaged from a single clone ancestor for 15 experimental host generations through nematode hosts under one of two different selection regimes: (i) SE repeated passage of *E. faecalis* alone in *C. elegans* and (ii) CCE repeated passage of *E. faecalis* in *C. elegans* with a fixed, non-evolving *S. aureus* isolate. In treatment (i), nematodes were only exposed to *E. faecalis*, while in (ii), nematodes were exposed to *E. faecalis* first, so that the microbe could establish residency, and then to *S. aureus*. We enforced within-host interactions between the bacterial species by propagating *E. faecalis* cells harvested from bacteria-killed nematodes, a method that also avoided direct selection against virulence and for host health. All replicate populations were passaged at the same time during the experiment.

Here, we use experimental evolution to test whether a mildly pathogenic, resident microbe (*Enterococcus faecalis*) can evolve to defend its host (*Caenorhabditis elegans*) against infection by a more virulent pathogen (*Staphylococcus aureus*), and thus cross the parasitism–mutualism continuum. *E. faecalis* and *S. aureus* frequently occur in animal and human microbiomes (Holden *et al.*, 2004; Martin-Vivaldi *et al.*, 2010; Lawley *et al.*, 2012; Cruz *et al.*, 2013; Kommineni *et al.*, 2015) wherein they can be pathogenic or commensal. Both bacteria can colonise the gut of *C. elegans* (Garsin *et al.*, 2001), a model animal system for investigating natural and lab-based host–microbiota associations (Cabreiro and Gems, 2013; Clark and Hodgkin, 2013; Petersen *et al.*, 2015) and their evolutionary consequences (reviewed in Gray and Cutter, 2014). Within the lifetime of an individual nematode, both *S. aureus* and *E. faecalis* can be harmful. *S. aureus* is highly virulent, causing 100% host mortality after approximately 2 days of exposure (Sifri *et al.*, 2003) by lysing the cells lining the gut wall of nematode hosts (Gravato-Nobre and Hodgkin, 2005). By contrast, *E. faecalis* is lethal to *C. elegans* only over the longer term, requiring more than 7 days of exposure (and no food) to cause total host population reduction (Sifri *et al.*, 2002) and is only mildly pathogenic in shorter-term infections. In our experimental set-up, involving 2-day colonisations (described in Figure 1), *S. aureus* is a highly virulent pathogen in single infection, whereas *E. faecalis* is a mildly pathogenic resident of the nematode gut, causing c. <1% mortality of the host. *E. faecalis* is under selection in this state.

We tested whether *E. faecalis* resident within *C. elegans* evolved to protect against *S. aureus* infection where its host was challenged with the pathogen over 15 experimental host generations. Our experiments examined the following interaction: resident *E. faecalis* was allowed to evolve inside hosts in the presence/absence of a genetically fixed pathogen (supplied from ancestral culture each host generation; experimental procedure in Figure 1), and the properties of *E. faecalis* were compared between the two treatments. Both treatments consisted of six replicate populations started from a single clone of *E. faecalis* that were then independently passaged, and thus any adaptive evolution that occurred was due to *de novo* mutation and selection. We passaged *E. faecalis* from dead hosts to observe evolutionary processes arising from species interactions within hosts, rather than imposing direct selection for host health. We found that host protection against *S. aureus* by resident *E. faecalis* evolved rapidly within nematode hosts in all replicate populations. Genomic and subsequent biochemical analyses pointed to increased production of antimicrobial superoxide as the mechanism. Our results indicate that resident microbes, even mildly pathogenic ones, can rapidly evolve to defend their hosts in response to virulent pathogenic infection.

## Materials and methods

### Nematode host and bacteria

*C. elegans* is a nematode that constantly interacts with microbes in its natural habitat (Felix and Braendle, 2010), and it can act as a predator or host for numerous species (Cabreiro and Gems, 2013; Clark and Hodgkin, 2013; Petersen *et al.*, 2015). These animals are thus an established model for microbial colonisation and pathogenesis (Gravato-Nobre and Hodgkin, 2005) and their gut can be co-colonised by multiple pathogens and commensals (Peleg *et al.*, 2008; Portal-Celhay and Blaser, 2012; Montalvo-Katz *et al.*, 2013; Hodgkin *et al.*, 2013).

The N2 wild-type nematode strain used herein was obtained from the Caenorhabditis Genetics Center (University of Minnesota, Minneapolis, MN, USA). We used the *E. faecalis* lab strain OG1RF (Garsin *et al.*, 2001), an isolate from the human digestive tract, and *S. aureus* strain MSSA476 (Holden *et al.*, 2004), a disease-causing pathogen.

### Experimental evolution

A single, randomly selected clone of *E. faecalis* was the ancestor for all evolving populations, and stock of a single clone of *S. aureus* was used. Thus, only *E. faecalis* was permitted to evolve in response to species interactions whereby they inhabited the *C. elegans* gut alone (single evolution, SE) or with *S. aureus* (co-colonisation evolution, CCE; Figure 1). Nematodes also remained evolutionarily static throughout the experiment. A stock population of N2 wild-type nematodes was derived by isolating a single hermaphroditic female every generation from the population for five generations to ensure genetic homogeneity. Stock populations of the ‘isofemale' line were routinely maintained on nematode growth medium plates seeded with 50 ul of *Escherichia coli* OP50 in Luria-Bertani broth and kept at 20 °C. The nematodes digest *E. coli* after this bacterium is consumed, and it does not accumulate in the gut.

### Exposure, transfer and selection

Bacteria were cultured in Todd-Hewitt (TH) broth at 28 °C overnight. Lawns of *S. aureus* liquid culture (60 μl) were plated onto 9 cm petri plates with Tryptone Soy Broth (TSB) agar, and lawns of *E. faecalis* culture (60 μl) were also plated on TSB with 100 μg ml^−1^ rifampicin (in both experimental evolution treatments). This antibiotic is used to select for *E. faecalis* OG1RF from mixed cultures. Bacterial lawns were placed at 28 °C overnight and then cooled at room temperature for several hours prior to use.

For a given replicate, approximately 900 L4 (larval) individuals, previously feeding on *E. coli*, were transferred in M9 buffer to an exposure plate with *E. faecalis*. In the CCE treatment, after 24 h, all nematodes were washed off the plate with M9 buffer and centrifuged at 3000 r.p.m. for 3 min. The supernatant was discarded, and then 5 ml M9 buffer was added to the test tube. This washing procedure was repeated five times to clean excess bacterial cells off the nematode cuticle. Nematodes were in the M9 buffer for <10 min at any given point in time. Nematodes were then transferred to the second exposure plate with *S. aureus* from a frozen culture stock. During exposures, nematodes were placed at 25 °C. *E. faecalis* populations evolved in the absence of *S. aureus* during the SE treatment were simply maintained in *C. elegans* on their plate without transfer during that period.

Twenty-four hours later, 15 bacteria-killed nematode carcasses were picked from a single replicate population and placed in a 1.5 ml centrifuge tube with 1 ml M9 buffer. The tube was centrifuged at 3000 r.p.m. for 3 min, the supernatant was discarded, and 1 ml M9 buffer was added. The wash process was repeated five more times. After the final rinse, the nematode pellet was crushed with a pestle to release the pathogens from inside the carcass. The suspension was streaked onto selective media—TSB agar with 100 μg ml^−1^ rifampicin to isolate *E. faecalis*—and individual colonies were grown up at 28 °C overnight. Subsequently, 15 colonies of *E. faecalis* were picked from a given replicate population and mixed together in 5 ml THB overnight at 28 °C overnight. This liquid culture was then used to make a lawn for the next generation of exposures for that replicate. This procedure was identical for both experimental evolution treatments to control against possible impacts of rifampicin.

The liquid cultures of an ancestral colony (prior to selection) and evolved *E. faecalis* populations were frozen at −80 °C in 20% glycerol every five generations.

### Host mortality and bacterial fitness assays

Host mortality was assayed simultaneously for each population in each treatment at the end of the evolution experiment. We exposed approximately 200 L4 nematodes from the *C. elegans* stock to the ancestral and each of the 12 evolved populations of *E. faecalis* (from the G5, G10 and G15 experimental host generations). If populations were then tested with *S. aureus*, nematodes were washed off the *E. faecalis* exposure plate with M9 buffer into a 15 ml test tube, washed and transferred to the *S. aureus* exposure plate as described above. After 24 h of exposure, we counted the total number of dead nematodes. Nematodes were considered dead if they did not respond to touch with a platinum wire, as is standard in assays of *C. elegans* death. Simultaneously, approximately 200 nematodes were placed on each of six control plates with *E. coli* OP50, but no mortality was observed after 24 h. We also tested for within-population variation in the protective effect exhibited by CCE *E. faecalis*. Four colonies from each of the six replicate populations at G15 were randomly picked, separately grown in THB media and plated. Host mortality in the presence of *S. aureus* was tested as above.

We tested the generality of this protective effect against six pathogenic, genetically divergent *S. aureus* isolates (COL-MRSA, MSSA SH-1000, Newman, N13-MSSA, Mu50 MRSA, MRSA 252), in addition to MSSA476. All isolates were cultured the same way as described above. Similar to the methods above, approximately 50 L4 nematodes from the *C. elegans* stock were exposed to only *S. aureus*, or initially to populations of *E. faecalis* (ancestral or CCE G15) and then to *S. aureus*. After 24 h of exposure at 25 °C, we counted the total number of dead nematodes.

To examine the fitness differences of *E. faecalis* (ancestor vs SE at G15 vs CCE at G15) in co-colonised nematode hosts with *S. aureus*, we measured the number of colony-forming units (cfus) of *E. faecalis* and *S. aureus*. Five dead nematodes were picked from a plate, placed into 1 ml M9 buffer and washed repeatedly. After the final rinse, the nematode pellet was crushed with a pestle to release the bacterial cells from inside each carcass. The mixture was spread onto selective media to separate *E. faecalis* and *S. aureus* colonies (TSB with 100ug ml^−1^ rifampicin and Mannitol Salt Agar, respectively), and colonies were counted.

### Mechanism of pathogen suppression

#### Genomic analysis

To investigate the genetic basis of host protection conferred by *E. faecalis*, whole-genome resequencing was used for a randomly selected *E. faecalis* clone from each replicate at G15. The phenotype of that clone was confirmed as being consistent with population-level effects on nematodes as assessed above. DNA was isolated using either a DNeasy blood and tissue kit using standard methods for Gram-positive bacteria or using a modified CTAB extraction (von der Schulenburg *et al.*, 2001), and importantly, the addition of 10 mg ml^−1^ of Lysozyme (for *E. faecalis*) or Lyostaphin (for *S. aureus*) to the digestion step in both protocols was required. Illumina (San Diego, CA, USA) TruSeq Nano libraries were prepared from 200 ng of DNA according to the manufacturer's protocol and 250 bp paired-end reads generated on an Illumina MiSeq using v2 chemistry. Reads were trimmed for the presence of Illumina adaptor sequences using Cutadapt v1.2.1 and for a minimum quality score of Q20 using Sickle v1.200. The resulting reads (between 395 Mb and 715 Mb per sample) were then mapped to either *E. faecalis* OGIRF (NC_017316) or *S. aureus* MSSA4776 (NC_002953.3 and NC_005951, for main chromosome and plasmid, respectively) using BWA-MEM, duplicate reads were removed using Picard, local realignment and single nucleotide polymorphism calling was performed in GATK and structural variants detected using Breakdancer. Variants found in experimental but not ancestral clones were identified, and SnpEFF was used to predict their functional effects.

The genes with revealed single nucleotide polymorphisms were identified in the SEED database by ‘blasting' the corresponding sequences against a collection of *E. faecalis* genomes. The gene annotations were confirmed or suggested by analysis of the sequences and the 20000 bp window neighbourhoods of the corresponding genes. The composition of gene loci of the top 10 homologues in other bacteria was also analysed. STRING database and software was used to reconstruct gene connectivity networks for the detected genes. This application automatically assembles the data on gene positional associations in genomes, genetic, regulatory and physical protein interactions for the input genes that satisfy a set of confidence thresholds.

#### *In vitro* biochemical assays

We assessed for a difference in the ability of ancestral and evolved *E. faecalis* to produce superoxides as the mechanism of host protection. Ancestral *E. faecalis,* SE *E. faecalis* (at G15) and CCE *E. faecalis* (at G5 and G15) were grown overnight to stationary phase in TSB. Wells in an opaque, black 96-well plate with a transparent bottom were then inoculated with 5 μl from each overnight culture. Three technical replicates of each replicate population were made. Replicate populations that failed to grow properly in liquid culture were excluded from the analysis. The wells were prepared with 95 μl TSB and 100 μl of a reaction mixture from a superoxide ion assay kit (Sigma Aldrich, St Louis, MO, USA) containing luminol: a reagent that becomes luminescent following oxidation by superoxide, allowing the quantitative and relativistic measure of superoxide production. The inoculated reaction mixtures were monitored over 10 h (for which the kit was optimised by Sigma-Aldrich) and measured every 2.5 min for both OD_600_ and luminescence in a Synergy 2 plate reader (BioTek, Winooski, VT, USA). The actual luminescence produced by a sample is sensitive to starting conditions as it is proportional to bacterial biomass concentration. Bacterial growth is sensitive to several factors (that is, media concentration, population size) and is exponential, translating small differences in growth rate to large differences in luminescence. We thus simultaneously measured bacterial biomass concentration (OD_600_) and controlled for it in our luminescence data.

To examine the impact of superoxide production by evolved *E. faecalis* on *S. aureus* (and whether this was the source of suppression), we tested the degree to which the evolved enhanced suppression could be removed by the action of catalase (CAT) and superoxide dismutase (SOD). Superoxide dismutase converts superoxide into hydrogen peroxide, and CAT converts hydrogen peroxide into water and oxygen. Alone, SOD would remove superoxides by simply replacing it with harmful hydrogen peroxide. Likewise, CAT on its own would only remove the problems caused by hydrogen peroxide without affecting superoxides. Together, these enzymes create a pathway converting harmful superoxide into harmless products. If exogenous superoxides were responsible for *S. aureus* growth inhibition, we would therefore expect this inhibition to be lifted only when both enzymes are administered. Overnight cultures of all ancestral and CCE populations of *E. faecalis,* as well as *S. aureus*, were grown separately in TSB (standardised to an OD_600_ of 1.00±0.05). A solution of TSB was prepared with 0.25 M potassium phosphate buffer containing Superoxide Dismutase E.C. 1.15.1.1 (SOD) from bovine erythrocytes (Sigma-Aldrich) and Catalase E.C. 1.11.1.6 (CAT) from bovine liver (Sigma-Aldrich) each at 0.25 mg ml^−1^. An enzyme-free solution of TSB was also prepared as a control, containing the 0.25 M potassium phosphate buffer alone. The ancestor and CCE *E. faecalis* (two technical replicates of each replicate population) were mixed in equal ratios with *S. aureus*. From the liquid culture, 6 μl was used to inoculate wells in a 96-well plate with 196 μl of the TSB solution alongside wells of an *S. aureus* control (*S. aureus* only). The experiment was run in duplicate on the enzyme-free and enzyme-containing media. Cultures were shaken for 24 h at 30 °C, after which cfu counts were performed.

### Statistical analyses

All statistical analyses were conducted in SPSS 20.0.

#### Host mortality and bacterial fitness assays

Mortality data met assumptions of normality and equal variances. We performed an analysis of variance (ANOVA) on untransformed data to test for the difference in nematode mortality caused by *E. faecalis* and *S. aureus* independent colonisation, as well as their co-colonisation.

For the evolution experiment, we examined changes in nematode mortality every G5 in both the SE and CCE selection regimes. We performed a generalised linear model with binomial distribution (and maximum likelihood estimates) on host mortality data in the evolution treatments (with and without the presence of *S. aureus*) over time. Treatment and time (experimental generations) were fixed effects. A separate ANOVA was conducted to test for variation among isolates in within-population protective effects.

To examine the spectrum of host protection, we quantified nematode mortality upon co-colonisation by *E. faecalis* and a diverse range of *S. aureus* isolates including both laboratory and human disease isolates (Figure 4). An ANOVA was performed on host mortality data collected from single infections of *S. aureus* across the seven isolates to examine the variability in nematode mortality they produced. We then performed a generalised linear model with binomial distribution (and maximum likelihood estimates) on host mortality data with treatment (‘alone', ‘with ancestral *E. faecalis*' and ‘with CCE *faecalis*') and *S. aureus* isolate as fixed effects.

The number of *E. faecalis* and *S. aureus* viable cfus was square-root transformed to meet parametric assumptions. Separate ANOVAs were performed on transformed cfu values for each of *E. faecalis* and *S. aureus* within a dead, co-colonised nematode to test the effects of treatment on bacterial fitness. Least square mean contrasts were performed to test for differences between treatments.

#### Mechanism of superoxide production and pathogen suppression

Mean superoxide production was compared between ancestral and evolved *E. faecalis* populations from the *in vivo* experiment during the exponential growth phase (6–10 h) of the bacteria using *t*-tests as the data met assumptions of normality. Luminescence measurements were controlled for OD_600._

*S. aureus* growth in liquid culture was compared in the presence and absence of *E. faecalis*. We performed a generalised linear model with Poisson loglinear model (and maximum likelihood estimates) on *S. aureus* cfu values with treatment (‘alone', ‘with ancestral *E. faecalis*' and ‘with CCE *E. faecalis*'), and enzymes (presence and absence) as fixed effects. Their interaction was also evaluated.

## Results

### Changes in host mortality due to within-host microbial evolution

**Figure 2:**
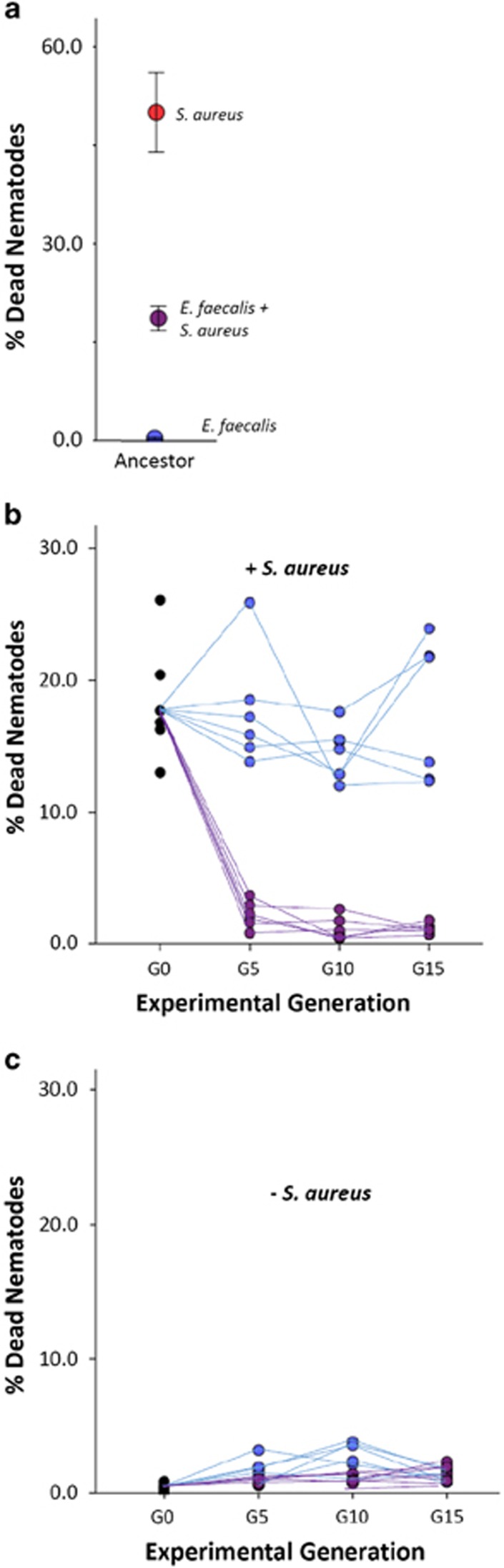
Effects of resident microbes on hosts over evolutionary time. (**a**) Host mortality with ancestral *E. faecalis* (blue circle) and *S. aureus* (red circle) separate and co-colonising (purple circle) in the nematode. The intermediate level of virulence from co-colonising bacteria species suggested the potential for *E. faecalis* to suppress pathogenic *S. aureus*. Error bars, 1 s.e. (**b, c**) Populations of *E. faecalis* were evolved under two different selection regimes: SE and CCE for 15 experimental host generations. To assess the ability of *E. faecalis* to protect hosts from *S. aureus*, host mortality in the (**b**) presence and (**c**) absence of *S. aureus* was quantified every G5 for SE (blue circles) and CCE (purple circles) *E. faecalis*. Lines connect each of the six replicate populations per treatment across time.

Ancestral *E. faecalis* is mildly pathogenic within the 2-day exposure window of this experiment, with <1% of nematodes dying after colonisation. In contrast, 52% of worms exposed to *S. aureus* died after exposure. Colonisation of worms with *E. faecalis* before exposure to *S. aureus* results in intermediate rates of nematode mortality, indicating that resident *E. faecalis* has the potential to suppress *S. aureus* virulence (Figure 2a; ANOVA: *F*_2,18_=51.908, *P*<0.001).

At the end of the evolution experiment, we assayed the protective ability of *E. faecalis* evolved in nematodes, either alone or with *S. aureus* co-colonisation (Figure 1). All of the six replicate CCE *E. faecalis* populations evolved to further suppress its virulence. Whereas 18% of worms died within 24 h of *S. aureus* exposure in the presence of the ancestral *E. faecalis* resident, this declined to 1% mortality, on average, in the presence of resident CCE *E. faecalis* from G5 onwards (Figure 2b). Although there is some among-population variation in the mortality rates caused by SE *E. faecalis* upon challenge with *S. aureus*, none of the replicate populations evolved significantly enhanced protective ability relative to the ancestor (Figure 2b; Generalised Linear Model, Treatment: Wald χ^2^=280.723, *P*<0.001; Time: Wald χ^2^=97.230, *P*<0.001). When four colonies within each replicate population of the CCE treatment were tested at G15, an equivalently enhanced protective effect was observed (ANOVA: F_5,24_=0.318, *P*=0.895).

Lower host mortality on *S. aureus* exposure was not associated with a reduction in *E. faecalis* virulence. Rather, whilst mortality remained generally low (<2%) in all replicate populations, *E. faecalis* evolved in both treatments to increase nematode mortality over time when tested alone (Figure 2c; Generalised Linear Model: Treatment: Wald χ^2^=9.126, *P*=0.003; Time: Wald χ^2^=22.510, *P*<0.001). Thus, although highly beneficial to hosts when tested in the presence of *S. aureus*, on its own, CCE *E. faecalis* remained mildly pathogenic and costly for the nematode host to possess. This result clearly demonstrates the context dependence of the fitness effects of this protective microbe upon hosts (Chamberlain *et al.*, 2014).

### Spectrum of host protection

**Figure 3:**
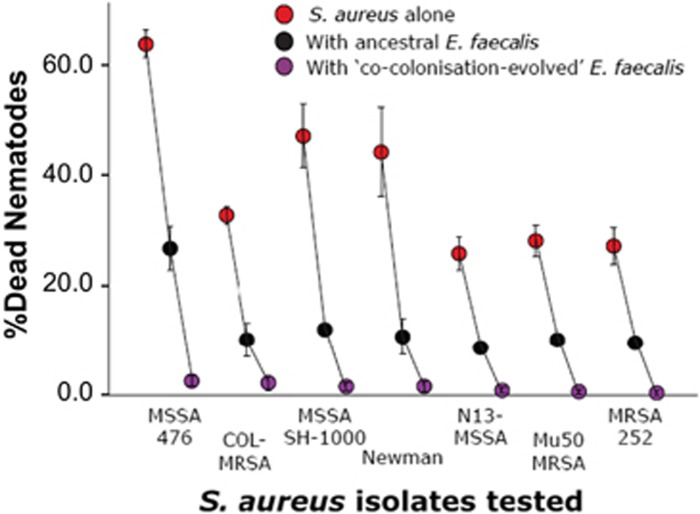
Generality of host protection by evolved *E. faecalis* against seven *S. aureus* isolates. Host mortality was evaluated after 24 h of exposure to *S. aureus*. Nematodes were exposed to *S. aureus* alone (red circles) or were previously colonised by ancestral *E. faecalis* (black circles) or CCE *E. faecalis* at G15 (purple circles). MSSA476 was used in the evolution experiment. Error bars, 1 s.e.

All CCE *E. faecalis* populations at G15 were effective at protecting nematode hosts against a broad spectrum of *S. aureus* isolates (Figure 3). In single infections, these *S. aureus* isolates exhibited variation in their virulence towards *C. elegans* (Figure 3; 26–65% mean nematode mortality; ANOVA *F*_6,42_=10.505, *P*<0.001). In co-colonised hosts, all *S. aureus* isolates with ancestral *E. faecalis* produced intermediate rates of host mortality, whereas with all replicate populations of CCE *E. faecalis*, nematode mortality was dramatically reduced to 0–1%. Both treatment and isolate affected the virulence of pathogens on hosts (Generalised Linear Model, Treatment: Wald χ^2^=370.961, *P*<0.001; Isolate: Wald χ^2^=303.650, *P*<0.001).

### Host protection and microbial growth within hosts

**Figure 4:**
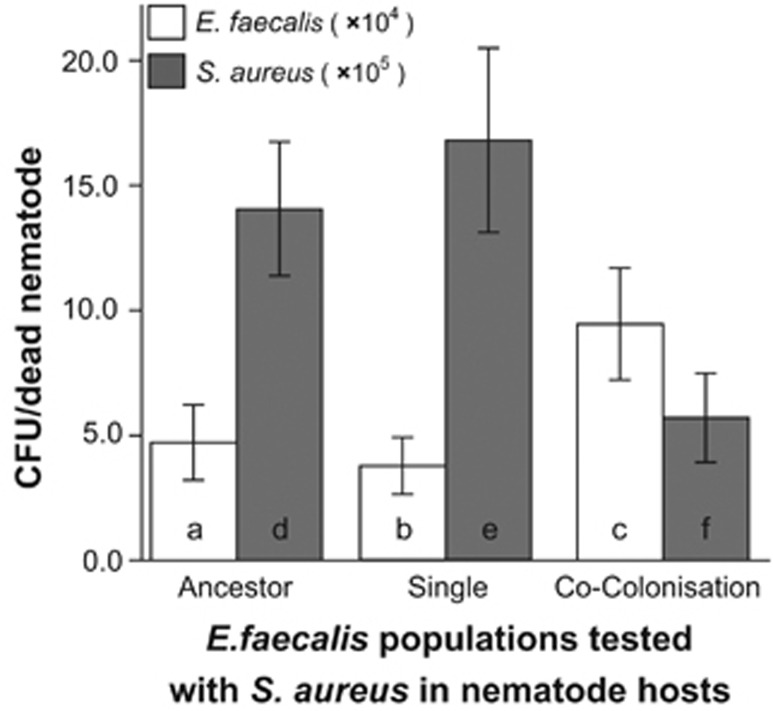
Fitness (cfus/nematode) of resident *E. faecalis* populations and infecting *S. aureus. S. aureus* is co-colonising with ancestral, SE, or CCE *E. faecalis* populations. Error bars, 1 s.e.

We examined whether the evolved *E. faecalis* suppression of *S. aureus* virulence was associated with increased *E. faecalis* proliferation and reduced *S. aureus* growth (Figure 4). Compared with ancestral *E. faecalis,* CCE *E. faecalis* (assayed at G15) suppressed *S. aureus* viable bacterial counts more (Figure 4; d vs f) and accumulated marginally more within nematodes (Figure 4; a vs c). By contrast, SE *E. faecalis* populations did not grow to higher density on average than the ancestor when interacting with *S. aureus* (Figure 4; a vs b). These SE populations were also associated with higher within-host growth of *S. aureus* compared with CCE *E. faecalis* (Figure 4; Analysis for *S. aureus* cfu: ANOVA across the three treatments: *F*_2,18_=4.072, *P*=0.039; Least Square difference d>f, *P*=0.038; Least Square difference e>f, *P*=0.019; Analysis for *E. faecalis* cfu: ANOVA across all three treatments: *F*_2,18_=3.603, *P*=0.053; Least Square difference a<c, *P*=0.057; b<c, *P*=0.023 a=b, *P*=0.649).

**Figure 5:**
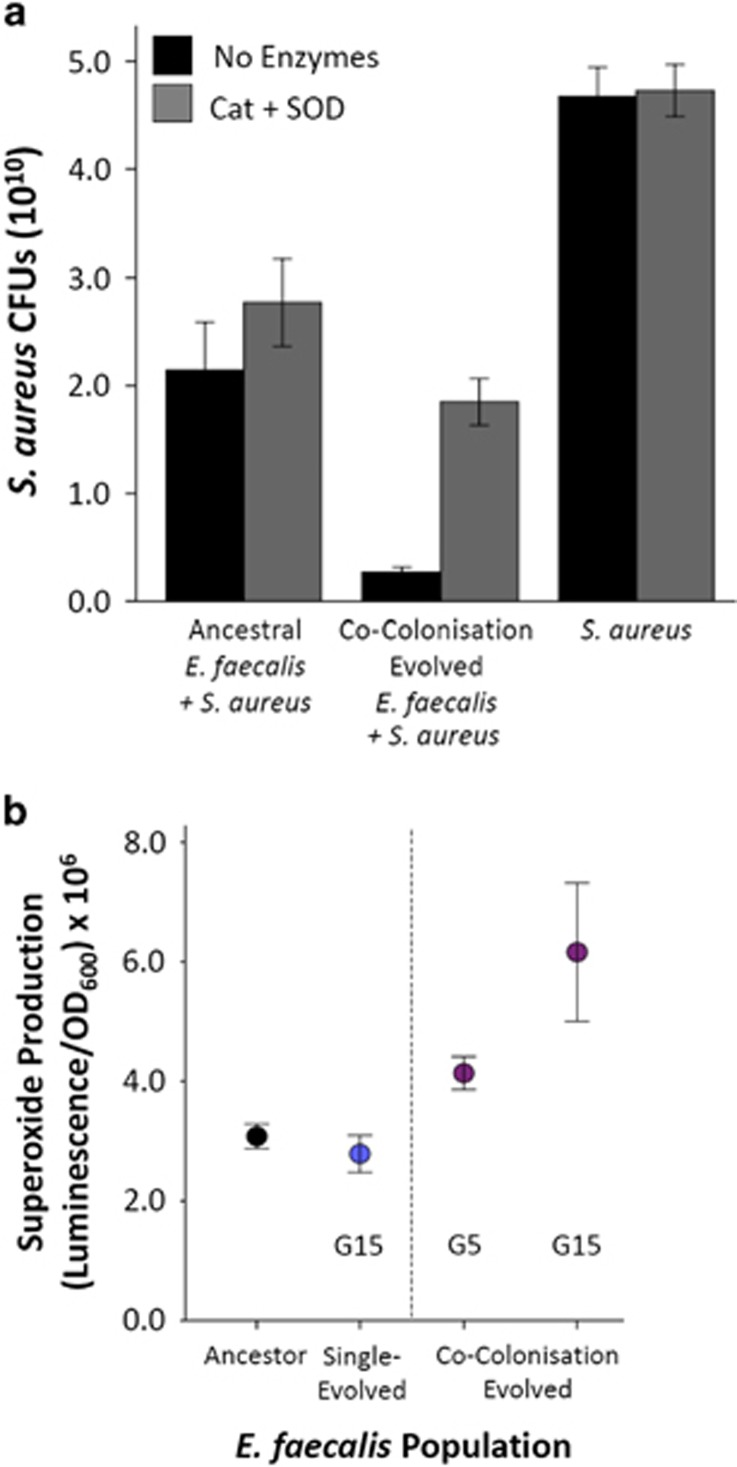
Evolved mechanism of suppression of *S. aureus* by *E. faecalis*. (**a**) Suppression and enzyme-mediated lifting of suppression of *S. aureus* outside the host. *S. aureus* cfus were counted when the pathogen was grown alone and co-cultured with ancestral or CCE *E. faecalis*. Counts were also made upon the addition of CAT and SOD, enzymes that remove the presence of reactive oxygen species. Error bars, 1 s.e. (**b**) Mean superoxide production (measure of luminescence controlling for OD_600_) across exponential growth phase of ancestral, SE and CCE *E. faecalis* (the latter at G5 and G15).

Suppression of *S. aureus* may occur either directly from the presence of *E. faecalis*, be mediated by *E. faecalis*-induced alterations to host biology or be a product of both direct and host-mediated effects. We assessed the importance of direct suppression using *in vitro* experiments. *In vitro* experiments recapitulated *in vivo* assays showing that CCE *E. faecalis* populations were better able to suppress *S. aureus* growth in liquid culture than ancestral *E. faecalis* (Figure 5a; Generalised Linear Model, Treatment: Wald χ^2^=3.18 × 10^11^, *P*<0.001).

### Genomic and biochemical analysis of the mechanism underpinning protection

To investigate the genetic basis of *E. faecalis*-mediated protection, we whole-genome resequenced a randomly picked clone of ancestral *E. faecalis* and evolved *E. faecalis* from each of the 12 replicate populations at G15. Each evolved *E. faecalis* clone exhibited a unique set of between one and three mutations (Supplementary Table 1). Consistent with the distinct phenotypes that evolved under the two contrasting treatments, the SE and CCE regimes selected for substitutions in different, functionally distinct gene sets. Six of 12 mutations in the CCE *E. faecalis* clones—1 per clone per replicated population—were putatively associated with superoxide production, but no mutations associated with this pathway were observed in clones from the SE treatment. *E. faecalis* is known to produce extracellular superoxide (Huycke *et al.*, 2011), mediated by dehydrogenase and fumarate reductase. Mutations were found in two NADH dehydrogenases and four genes associated with the respiratory complex function or purine biosynthesis. Purine biosynthesis represents the major pathway for fumarate production which, if blocked, leads to superoxide production (Supplementary Table 1; Supplementary Figure 1).

We therefore hypothesised that enhanced production of antimicrobial reactive oxygen species was the mechanism behind *E. faecalis*-mediated defence. In accordance with this hypothesis, all CCE *E. faecalis* populations produced more superoxide per cell than the ancestor, in both the G5 and G15 generations (Figure 5b; *t*-test: Ancestor vs G5, *t*=−3.056, df=31.385, *P*=0.005; Ancestor vs G15, *t*=−2.619, df=14.888, *P*=0.019). Moreover, there was no difference in superoxide production between SE and ancestral *E. faecalis* (*t*=0.788, df=20.329, *P*=0.440) suggesting that this trait only evolved during *S. aureus* challenge. The addition of CAT and SOD enzymes to growth media ablated the suppression of *S. aureus* growth by *E. faecalis* during *in vitro* interactions (Figure 5a; Generalised Linear Model, Enzymes: Wald χ^2^=8.49 × 10^10^, *P*<0.001), and had a greater effect at reducing suppression during interactions with CCE *E. faecalis* compared with the ancestor (Generalised Linear Model, Treatment × Enzymes: Wald χ^2^=7.24 × 10^10^, *P*<0.001). Together these data strongly point to increased superoxide production by evolved CCE *E. faecalis* as the mechanism of suppression of *S. aureus*.

## Discussion

The role of microbes in protecting their host against virulent pathogens has traditionally focused on ecological sources of protection, namely niche occupancy and competition for resources (for example, in insects, Gerardo and Parker, 2014). We hypothesised that owing to the high evolutionary potential of microbes—associated with their short generation times and large within-host population size—rapid *de novo* microbial evolution could have a role in shaping host resistance against infection. We observed the evolution of host-protective effects during microbial experimental evolution within nematode hosts in all independently passaged CCE populations, thus confirming the potential for this process to occur. Thus, *E. faecalis*, a microbe that has been observed in natural microbiomes to possess protective traits (Martin-Vivaldi *et al.*, 2010; Kommineni *et al.*, 2015), evolved across the parasite-mutualist continuum to become a host-protective mutualist upon pathogen attack. Notably, these host-protective effects evolved without any direct selection against host mortality. Instead, a beneficial relationship between the host and the resident bacterium emerged out of interactions with a virulent pathogen and selective processes acting upon the resident microbial populations. Although CCE *E. faecalis* populations evolved the ability to attenuate the high mortality caused by *S. aureus*, they also retained mild pathology against *C. elegans* when colonising alone, demonstrating the context dependence of their fitness effects on the host (Chamberlain *et al.*, 2014). In an environment where such virulent infection is common, *E. faecalis* would therefore now represent a net mutualist with respect to its impact on host fitness. This result reflects observations of other protective microbes found naturally, which defend their host whilst retaining pathogenicity (Martinez *et al.*, 2014; Polin *et al.*, 2014).

The mechanisms of microbe-mediated protection observed in nature are remarkably diverse (Gerardo and Parker, 2014). Although niche occupation (Dillon *et al.*, 2005), resource competition and immune system mediation (Abt and Artis, 2013; Hooper *et al.*, 2012; McFall-Ngai *et al.*, 2013) may still have a role in our system, the genomic evidence indicates that selection acted predominantly through direct *E. faecalis–S. aureus* interactions during host colonisation. Further experiments, however, are required to determine whether the microbial interactions observed to evolve here are adaptive to the host environment or whether similar evolutionary outcomes would arise *in vitro*. Regardless, we observed parallel evolution of the superoxide production pathway in CCE *E. faecalis* across all replicate populations, and we were able to ablate the evolved suppression through enzymatic treatment to remove superoxide radicals. These data strongly suggest that antimicrobial superoxides, which may act to directly suppress *S. aureus* or act indirectly via oxidation of the *S. aureus* auto-inducing pheromone (Rothfork *et al.*, 2004), are a key mechanism in the evolved protective phenotype. The lack of genotype specificity we observed is also consistent with a superoxide-mediated suppression system, which represents a broad-spectrum form of microbial suppression. While *C. elegans* itself produces reactive oxygen species in response to pathogen infection (Chávez *et al.*, 2007), reactive oxygen species produced by resident bacteria may also be a common means of broad-spectrum protection against infection, and one that is thus likely to be evolutionary labile in its activity. For instance, lactic acid bacteria in the guts of honeybees are able to suppress a range of pathogens, including *S. aureus* and *Pseudomonas aeruginosa* via reactive oxygen species production (Olofsson *et al.*, 2014). That our experimental treatment drove the evolution of a broad-spectrum defence mechanism, as opposed to more specific mechanisms of suppression (for example, bacteriocin secretion), is also consistent with observations from natural disease systems showing that microbes can protect against a diversity of pathogen isolates (Koch and Schmid-Hempel, 2011) and species (Martinez *et al.*, 2014).

The extent of the protective phenotype that evolved here, and the rate at which it evolved, were striking. Despite being regularly attacked by pathogens, if nematode hosts were colonised by evolving *E. faecalis*, they were almost universally protected against pathogens that would otherwise quickly kill most of the population. Moreover, although the evolution in our experiment occurred during passage through a number of individual worms, the time frame for the evolution of protection by *E. faecalis* was just 5 days of co-colonisation. This short timescale presents the possibility of the evolution of microbe-mediated protection within the lifetime of a longer-lived host, such as a mammal or tree, in which evolution is likely potentiated by larger population sizes.

Future research will need to establish how within-host evolution of microbial species would alter disease progression. A key simplification in our experiment is that the virulent pathogen is genetically fixed, thus mimicking a spillover zoonotic infection whereby the pathogen normally circulates in a different host species. An example is *Salmonella*. Some isolates of this bacterium commonly reside within the microbiomes of livestock animals, but can cause serious infections if transmitted to humans. Within a host individual, however, it is possible that pathogen evolution would also occur on a similar timescale, obviating any evolved protective abilities in resident microbial species and setting the stage for coevolutionary interactions. Our experiment also considers only a binary microbial interaction, whereas natural microbial communities are often highly diverse. The impacts of the evolution of the microbiome on pathogen attack (Mueller and Sachs, 2015) and on interactions within the microbiome also warrant consideration. Notwithstanding this, the potential for evolution of interactions between resident microbes and pathogens is clear, and future research on microbiome–pathogen relationships should go beyond ecological responses to examine evolved ones.
