# Supplementary material for: Rapid evolution of microbe-mediated protection against pathogens in a worm host
Source: ISME J. 2016 Mar 15;10(8):1915–24. doi: 10.1038/ismej.2015.259 (PMC5029159; doi:10.1038/ismej.2015.259)
Supplement: Supplementary Material [file ismej2015259x1.doc]

**Supplementary Materials:**

**Figure 1**

**Table 1**

**
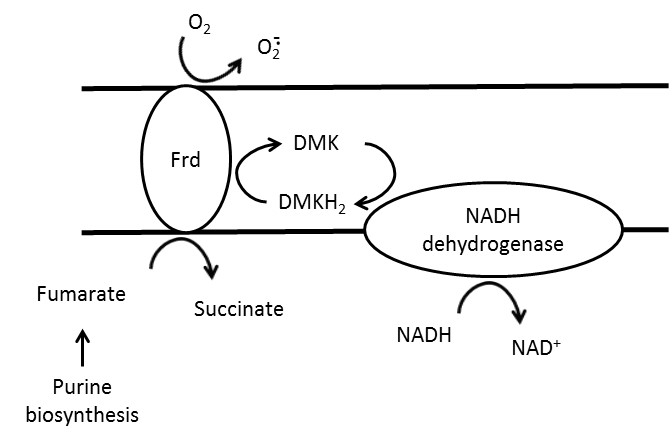
**

Supplementary Figure 1: Schematic of extracellular superoxide production (O_2_-) in *Enterococcus faecalis*. In mitocondrial respiration and sugar fermentation, electrons flowing through NADH can naturally react with oxygen to produce low levels of extracellular superoxide. In *E. faecalis*, these pathways have evolved to produce substantial amounts of superoxide. When fumarate is unavailable as a terminal electron acceptor, superoxide production is promoted by exposure of oxygen to reduced demethylmenaquinone (DMKH_2_) mediated by fumerate reductase (Frd). Model based on Huycke et al.

Supplementary Table 1: Location of mutations in experimentally evolved *E. faecalis* populations and their putative functions.

| Sample (Treatment, Population) | SNP position | Mutation effect | Gene | Putative function |
| --- | --- | --- | --- | --- |
| Co-Colonisation 1 | 1617044 | stop gained | OG1RF_11546 | Phosphoribosylaminoimidazole carboxylase, purine biosynthesis |
| Co-Colonisation 1 | 2305950 | stop gained | OG1RF_12189 | MarR transcriptional regulator, bacterial detoxification, positionally associated with purine biosynthesis in several Gram positive bacteria |
| Co-Colonisation 2 | 2136704 | downstream | OG1RF_12020 | Positionally clustered to Fumarate utilization gene clusters in Lactococci |
| Co-Colonisation 2 | 2572990 | non-synonymous | ymdA | Ribonuclease Y |
| Co-Colonisation 3 | 1362213 | non-synonymous | ndh | NADH dehydrogenase |
| Co-Colonisation 3 | 1817527 | frameshift | rmlA | Glucose-1-phosphate thymidylyltransfer |
| Co-Colonisation 4 | 91045 | non-synonymous | OG1RF_10084 | Cell surface wall protein |
| Co-Colonisation 4 | 649795 | frameshift | OG1RF_10613 | Positionally clustered to fumarate reductase flavoprotein subunit in *Pediococcus* |
| Co-Colonisation 4 | 1729495 | non-synonymous | ndh2 | NADH dehydrogenase |
| Co-Colonisation 5 | 1155243 | non-synonymous | ftsK | Cell cycle protiein, associated with ndh2 in string database |
| Co-Colonisation 6 | 2162278 | downstream | OG1RF_12043 | Dithiol-disulfide isomerase |
| Single 1 | 264367 | downstream | OG1RF_10252 | Clusters to sugar utilisation /catabolic genes |
| Single 1 | 1718052 | non-synonymous | comG2 | Positionally associated with Glycine cleavage system |
| Single 1 | 2287392 | downstream | OG1RF_12166 | Clusters to vitamin B12, Choline, betaine-related functions, glycolysis, isoprenoid biosynthesis, glucoseamines |
| Single 2 | 616393 | non-synonymous | pip | clusters to sugar metabolic genes. |
| Single 2 | 2572903 | non-synonymous | ymdA | Ribonuclease Y |
| Single 3 | 2513320 | non-synonymous | OG1RF_12374 | Clusters with glucose-1-p-thymidylyltransferase in *L. lactis* |
| Single 4 | 2221444 | downstream | OG1RF_12100 | Exonuclease, pyrimidine metabolism |
| Single 4 | 2514210 | downstream | OG1RF_12376 | Hypothetical protein |
| Single 5 | 61667 | frameshift | OG1RF_10056 | 5’ nuclease |
| Single 5 | 2573805 | downstream | OG1RF_12442 | Rrf2-linked NAD-FMN reductase |
| Single 6 | 1447813 | non-synonymous | cryZ | Agrinate lyase |
| Single 6 | 2513320 | non-synonymous | OG1RF_12374 | Hypothetical protein |
